# LOS ANGELES COUNTY BOLD INITIATIVE: LAYING THE GROUNDWORK FOR STRATEGIC PLANNING ON DEMENTIA

**DOI:** 10.1093/geroni/igac059.1761

**Published:** 2022-12-20

**Authors:** Tony Kuo, Noel Barragan, Mariana Reyes, Mateeha Zafar, Cinthie Lopez Paz, Ariane Thomas

**Affiliations:** University of California, Los Angeles, Los Angeles, California, United States; Los Angeles County Department of Public Health, Los Angeles, California, United States; Los Angeles County Department of Public Health, Los Angeles, California, United States; Los Angeles County Department of Workforce Development, Aging and Community Services, Los Angeles, California, United States; Los Angeles County Department of Workforce Development, Aging and Community Services, Los Angeles, California, United States; Los Angeles County Department of Public Health, Los Angeles, California, United States

## Abstract

Funded by the Centers for Disease Control and Prevention’s Building Our Largest Dementia Public Health Program through U.S. Congressional appropriations for the BOLD Infrastructure for Alzheimer’s Act, the Los Angeles County BOLD initiative (LA BOLD) was established in 2021 with the goal of addressing the silent but burgeoning epidemic of Alzheimer's disease and related dementias (ADRD) in Los Angeles County (LAC). In 2019, approximately 166,857 LAC residents were living with Alzheimer’s disease; this number is expected to increase 150% by 2040. The initiative brings together a diverse group of multi-sector stakeholders, including two Area Agencies on Aging, two major academic centers, the largest Medicaid health plan in the region, the county’s safety net health agency (Public Health, Health Services, Mental Health), and other key community partners. The primary objectives of LA BOLD are: (i) establish a countywide coalition to address ADRD locally, and (ii) develop a strategic plan to guide the coalition’s efforts. The present study describes the first year of this initiative, highlighting the formation of the coalition’s steering group, identifying the three focus areas for strategic planning – risk reduction, early detection, advance care planning – and sharing lessons learned from navigating the contextual realities of ADRD care and prevention in LAC’s diverse communities. Data and program accomplishments for this early phase of LA BOLD are discussed within the context of medical advancement, the financial realities of ADRD programming and services, and the geo-political factors that are being considered as part of the strategic planning and implementation processes.

